# Is something hidden during tachycardia?

**DOI:** 10.1007/s12471-016-0888-5

**Published:** 2016-09-01

**Authors:** D. Mol, M. T. Rijnierse, G. S. de Ruiter

**Affiliations:** 1 Department of Cardiology, OLVG, Amsterdam, The Netherlands; 2 Department of cardiology, VU University Medical Center, Amsterdam, The Netherlands

## Answer

**Fig. 1:**
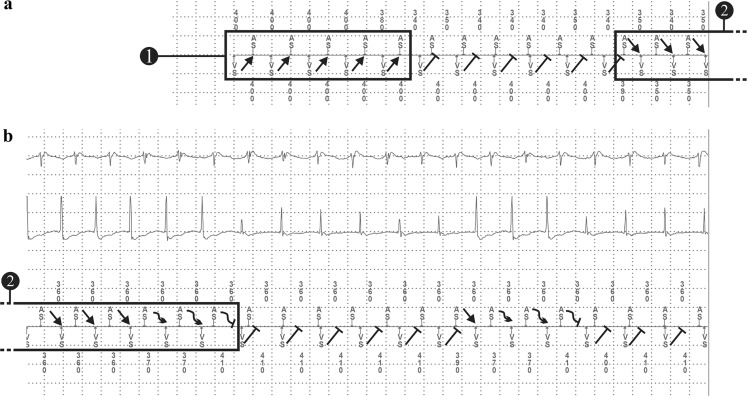
Intracardiac ICD recording prior to VT ablation; classified as supraventricular tachycardia

**Fig. 2:**
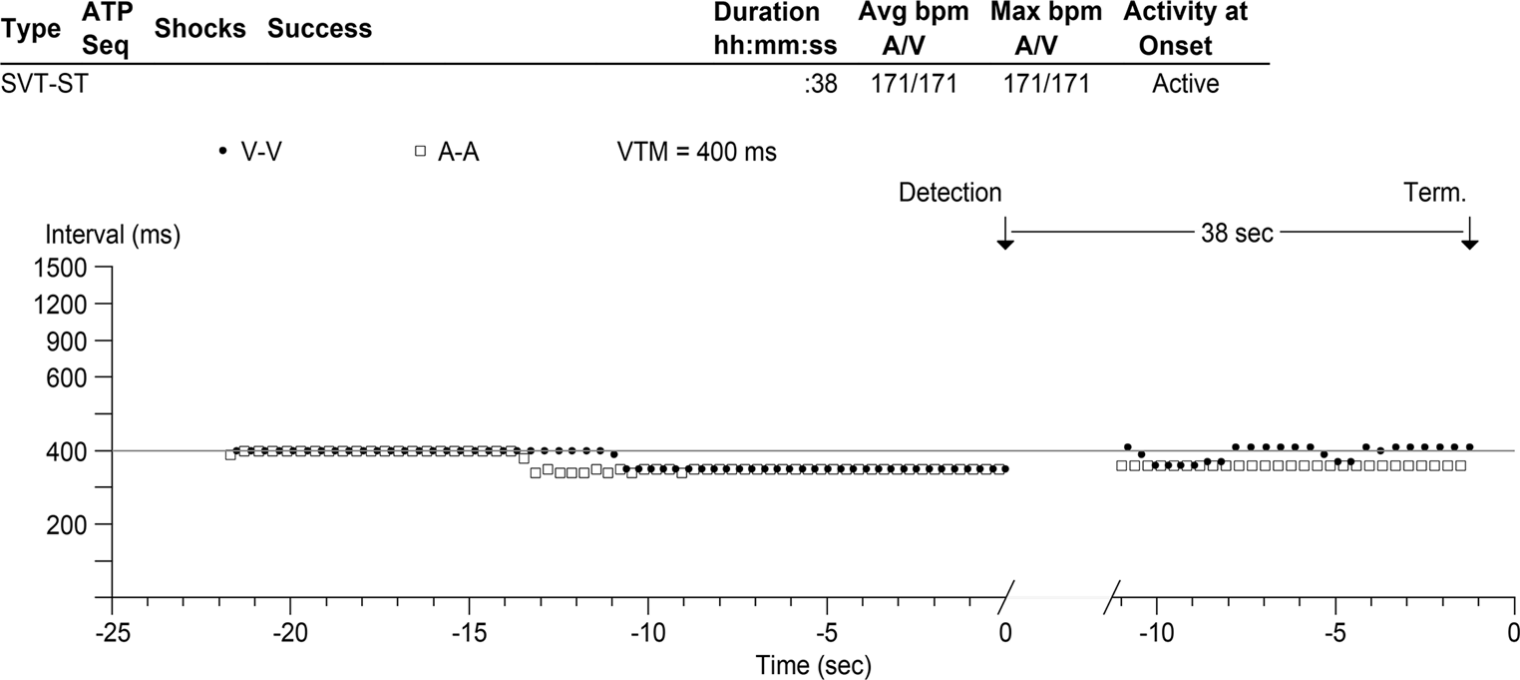
Plot diagram of the recorded ICD episode

Box 1 shows a ventricular tachycardia (VT) with 1:1 retrograde conduction to the atrium, cycle length (CL) 400–410 ms. Directly after Box 1 the retrograde conduction induces atrial tachycardia, CL 340 ms. Consequently, atrioventricular (AV) dissociation is present which favours the diagnosis of VT. The retrograde conduction of the ongoing VT results in a refractory AV node hampering the anterograde AV conduction of the induced atrial tachycardia. At the beginning of Box 2, the atrial tachycardia overdrives the VT because the atrial complex is timed when the AV node is excitable, resulting in anterograde conduction with acceleration of the ventricle. The second part of the tracing is demonstrated in Fig. 1b. The atrial tachycardia with 1:1 anterograde AV conduction is still present. After a few beats, AV conduction becomes decremental. This resulted in reappearance of the VT with similar CL 400–410 ms, suggesting the presence of concealed ongoing VT in the VT circuit during atrial tachycardia with 1:1 conduction as the VT was not terminated by the overdrive suppression of the atrial tachycardia. This may be explained by an intra-ventricular conduction delay and the long distance from the site of overdrive suppression (His bundle) to the circuit of the VT (apex). Resetting the VT by an activation with an origin far from the circuit needs a shorter CL to increase the possibility to enter the excitable gap of the re-entry circuit [1]. The phenomenon of Fig. 1a repeats with again decremental conduction after a few beats. The plot diagram (Fig. 2) gives a overview of both tachycardias.
